# Community Health Worker programmes’ integration into national health systems: Scoping review

**DOI:** 10.4102/phcfm.v15i1.3204

**Published:** 2023-03-09

**Authors:** Lucia M. Mupara, John J.O. Mogaka, William R. Brieger, Joyce M. Tsoka-Gwegweni

**Affiliations:** 1 Department of Public Health Medicine, College of Health Sciences, University of KwaZulu-Natal, Durban, South Africa; 2 Department of International Health, The Johns Hopkins Bloomberg School of Public Health, Johns Hopkins University, Baltimore, United States; 3 Faculty of Health Sciences, University of the Free State, Bloemfontein, South Africa

**Keywords:** community health work, Integration, CHW programmes, health systems strengthening, national health systems, improved health outcomes, Sub-Saharan Africa

## Abstract

**Background:**

Community health worker (CHW) programmes, when adequately integrated into mainstream health systems, can provide a viable, affordable and sustainable path to strengthened health systems that better meets demands for improved child health, especially in resource-constrained settings. However, studies that report on how CHW programmes are integrated into respective health systems in sub-Saharan Africa (SSA) are missing.

**Aim:**

This review presents evidence on CHW programmes’ integration into National Health Systems for improved health outcomes in SSA.

**Setting:**

Sub-Saharan Africa.

**Method:**

Six CHW programmes representing three sub-Saharan regions (West, East, and Southern Africa) were purposively selected based on their deemed integration into respective National Health Systems. A database search of literature limited to the identified programmes was then conducted. Screening and literature selection was guided a scoping review framework. Abstracted data were synthesised and presented in a narrative form.

**Results:**

A total of 42 publications met the inclusion criteria. Reviewed papers had an even focus on all six CHW programmes integration components. Although some similarities were observed, evidence of integration on most CHW programme integration components varied across countries. The linkage of CHW programmes to respective health systems runs across all reviewed countries. Some CHW programme components such as CHW recruitment, education and certification, service delivery, supervision, information management, and equipment and supplies are integrated into the health systems differently across the region.

**Conclusion:**

Different approaches to the integration of all the components depict complexity in the field of CHW programme integration in the region.

**Contribution:**

The study presents synthesized evidence on CHW programmes integration into national health systems in SSA.

## Introduction

Mortality among children under 5 years of age has long been a major public health problem.^1^ The United Nations Millennium Development Goals (MDGs) initiative facilitated the reduction of global under-five mortality rate (U5MR) from 91 deaths per 1000 live births in 1990 to 43 deaths per 1000 live births in 2015.^2^ This initiative is believed to have saved the lives of 48 million children under the age of 5 years during 2000–2015.^3^ Encouraging as this accomplishment sounds, the current global U5MR is still a cause for concern.

The World Health Organization (WHO) has expressed concern that more than half of the deaths in children under 5 years of age are attributed to preventable and treatable diseases or conditions,^3,4^ which can be addressed by an enhanced uptake of and access to an array of low-cost and modest community-based interventions for maternal and child health.^5,6,7,8,9^ These preventive community-based interventions can be delivered by community health workers (CHWs). However, the implementation of these community-oriented interventions in resource-constrained settings is inhibited because of weak health systems among other restraints.^5,7,8,10,11,12,13^ The use of CHWs to strengthen health systems has a long history and continues to be a contemporary discourse at many international public health platforms.

The WHO defined CHWs as:

[*M*]embers of the communities where they work, should be selected by the communities, should be answerable to the communities’ needs and priorities, should be supported by the health system but not necessarily a part of the government, and have shorter training than professional health workforce.^14^

The prospective to effectively render ‘cost-effective, high-quality and culturally competent health services within team-based care models’^15^ has catapulted CHWs to recognition by the WHO and the Global Health Workforce Alliance (GHWA) as an integral component of the health workforce needed for health system strengthening (HSS) to improve child health outcomes.^5,8,9,16,17,18,19,20,21^

There have been calls for this health workforce cadre to be integrated into National Health Systems.^9,22^ Evidence in existing literature points to the fact that CHWs’ full potential in strengthening health systems is maximised when optimally integrated into health system building blocks.^5,8,23,24,25,26,27^ The call for CHW integration was a response to prior research findings that, historically, ‘CHWs have been essentially at the margins of the health care system with reference to remuneration, professionalization and advocacy to mention a few, in most countries’.^5,12,28,29,30^

Although recent literature has defined context, mechanisms and guidelines for integrating CHW programmes into mainstream health systems,^5,8,9,10,11,16,17,26,27,31^ not many studies have assessed how CHW programmes are integrated into respective health systems in sub-Saharan Africa (SSA). As a result, the subject of CHW integration has become one of the biggest areas that need to be addressed, especially in this region. It is against this backdrop that this paper sought to review and synthesise the existing evidence on CHW programmes’ integration into National Health Systems at policy level in SSA. The scoping review serves as a foundation for future research into the gap between policy and practice, generating policy-level evidence that may influence implementation at the National Health Systems level. Community health worker integration policies are important because they help establish guidelines that benefit the community, individual patients, and the health system in general.^26^ Having protocols such as the ones assessed in this study can help prevent human error and poor communication around CHW programme decisions. For CHWs and the broader health workforce, understanding and following established CHW policies can help ensure that health care delivery at the community level is optimised, while practice feedback are used to help inform future CHW policies.

Policy processes are commonly depicted as linearly cyclic,^32^ comprising rationale formulation, objective setting, option assessment, monitoring and evaluation of effects and results, and feedback looping back into the cycle. However, research shows that there is a significant gap between policy and implementation.^33,34^ Many conditions, such as unanticipated budgetary restrictions, public opinion, political values and ideology, epidemiologic occurrences, and social and economic realities, frequently affect policies, which are not represented in the simple ways in which policies are constructed. Given the complexities of policy implementation, it is probable that not all of the CHW policies reviewed produce observable and traceable outcomes. The investigation of the policy implementation mismatch is however outside the scope of this study.

## Conceptual framework

In line with Mary Dixon-Woods’ suggestions,^35^ this study employed a conceptual framework to guide and organise data abstraction and analysis. While the concept of integration can refer to the mere combination of two or more existing but unrelated programmes, it can also focus on the introduction of new health cadres and programmes into the mainstream health system.^36^ According to Atun et al.,^37^ integration is ‘the process or extent and pattern of acceptability and adoption of the health intervention – in this case a CHW programme – into critical functions of a health system’. These critical functions include: service delivery, human resources for health, information, medical products, financing, and leadership and governance.^13^ To this effect, this study adapted the WHO framework for HSS^13^ to guide the search for evidence of integration of CHW programme components into respective building blocks. We acknowledge the critique of WHO’s building blocks framework because of its oversight on the interrelationships of several health system components, and the omission of customers and communities who are at the centre of health systems. Nevertheless, the use of the framework for this study was anchored on the fact that some of the misgivings have since been addressed in a reviewed edition of the framework.

**FIGURE 1:**
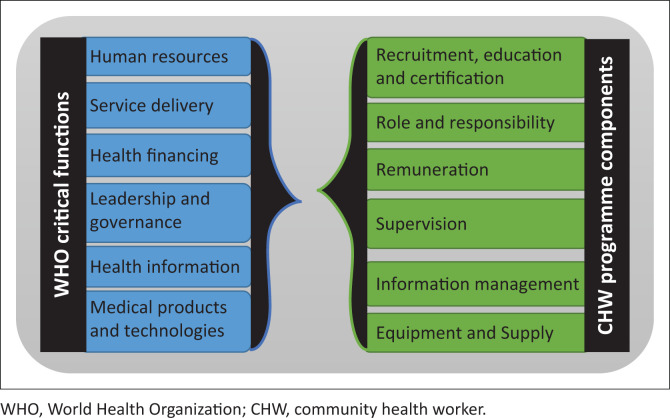
World Health Organization building blocks and corresponding community health worker programme components.

The evidence of integration sought for included government and/or Ministry of Health (MOH) standardised documented polices, plans and guidelines^38^ on each of the integration variables that seek to improve on the WHO health system building blocks. The integration variables chosen for this study are CHW recruitment, education and certification, CHW roles and responsibility, CHW documentation and information management, CHW remuneration, CHW supplies and equipment, and CHW supervision, respectively. The study therefore analysed each CHW programme component according to the elements of the WHO framework for HSS. Figure 1 shows how the proposed WHO building block maps onto the CHW programme components.

Conceptual framework used in this study shows WHO building blocks^13^ and corresponding CHW programmes’ integration variables^31^ at the community and national health system levels. The WHO’s set of six essential building blocks is mapped onto the six CHW programme components to ensure CHW service is availed to individuals and families at the community level, including efficient referral systems, increased use of preventive and lifesaving medical interventions. At the same time these 6 × 6 WHO-CHW variables must be matched to ensure that CHW governance and capacity issues are adequately addressed at national health system levels, such as higher levels of service coordination, interdepartmental policy and process planning. The conceptual framework for this study was adopted from Mupara et al.^39^

## Methods

### Study design

We conducted a systematic scoping review to provide evidence synthesis on how CHW programmes focusing on child health in SSA are integrated into National Health Systems. A scoping review or scoping study is:

[*A*] form of knowledge synthesis that addresses an exploratory research question aimed at mapping key concepts, types of evidence, and gaps in research related to a defined area or field by systematically searching, selecting, and synthesizing existing knowledge.^40^

The review was guided by Arksey and O’Malley’s scoping review framework.^41^ Preferred Reporting Items for Systematic Reviews and Meta-Analyses (PRISMA) guidelines were followed.^42^ The scoping review methodology was preferred because it is appropriate for attaining the objectives of this study which are to chart and synthesise evidence on what is published on integration of CHW programmes into health systems in SSA.

### Search strategy and eligibility criteria

Selection and classification of articles was informed by prior consultation with subject experts in the field of CHW programmes in SSA. This consultation resulted in the identification of candidate CHW programmes that are being operated as comprehensive programmes across several SSA countries. For the purposes of restricting our study to those CHW programmes that are known to be active and operated by respective central governments, a decision was then taken to (1) further consult documentary materials evidencing identified CHW programmes’ integration and (2) purposively sample identified CHW programmes to represent different SSA regions.^43^ Consequently, a pair of CHW programmes that address child health issues was selected to each represent the three sub-Saharan regions, namely West Africa (Ghana and Nigeria), East Africa (Ethiopia and Rwanda) and Southern Africa (Malawi and Zambia) (see Figure 3). These strategies finally resulted in the identification of the following six government run CHW programmes:

- Health extension workers (HEWs) in Ethiopia.
- Community health officers (CHOs) in Ghana.
- Health surveillance assistants (HSAs) in Malawi.
- Junior community health extension workers (JCHEWs), community health extension workers (CHEWs) and community officers (CHOs) in Nigeria.
- Agents de santé maternelle (ASM) and binomes in Rwanda.
- Community health assistants (CHAs) in Zambia.

It may appear that the use of purposive sampling in this study makes it difficult to demonstrate that the sample is representative of the wider population of interest. However, because the findings of a research based on purposive sampling can only be generalised to the subpopulation from which the sample is obtained and not to the entire population,^44^ the conclusions of this study are only generalisable to the selected category of CHWs – those run by central governments. Furthermore, the authors attempted to prevent bias in case selection by interviewing subject experts in the field of CHW programmes in SSA and extensively reviewing relevant documents on CHW programmes’ integration criteria. Researcher bias is only a real threat to a study’s credibility if the researcher’s judgements are poorly considered, or when they have not been based on clear criteria.^44^ Moreover, unlike in statistical considerations where the term ‘population’ refers to the entire group with the characteristics of interest,^45^ ‘population’ as used in this study only refers to a subpopulation of CHW programmes that are government-operated. This subgroup can therefore be said to be a representative of all similarly run CHW programmes in SSA (cases having been selected to represent the southern, eastern and west African regions). In other words, while the study findings were not generalisable as descriptions of generic CHW programmes in SSA, they were generalisable as descriptions of what any central government-run CHW programme can be, given that these CHW programmes have the same array of interlinking structures and policy projections as the ones surveyed in this study.

### Inclusion and exclusion criteria

Having selected the CHW programmes therefore, the following eligibility criteria guided further selection and classification of articles that were to be admitted into the study:

- Studies whose specific focus was on CHW programmes in any of the six selected SSA countries (as listed earlier).
- Studies that described the design and governance of the CHW programmes including government policy documents, CHW country profiles and Community Health Systems Catalogue (an online CHW resource pool that contains information on community health programmes, CHWs, and CHW interventions containing summaries of country-specific CHW programmes policies, strategies, guidelines, plans and reports based on the WHO health system framework)
- The studies had to be in English.
- The studies should have been published between January 2010 and December 2018.

### Search terms

Guided by the aforementioned criteria on how to admit eligible papers, we searched databases and grey literature repositories for works that evidenced how the selected CHW programmes are integrated into National Health Systems. Using specific CHW programme names as they were specifically referred to in the selected countries, we designed the search terms to include terms as follows:

- Health Extension Workers (HEWs) in Ethiopia,
- Community Health Officers (CHOs) in Ghana,
- Health Surveillance Assistants (HSAs) in Malawi,
- Junior Community Health Extension Workers (JCHEWs), Community Health Extension Workers (CHEWs) and Community Officers (CHOs) in Nigeria,
- Agents de santé maternelle (ASM) and Binomes in Rwanda, or Community Health Assistants (CHAs) in Zambia.

Databases that were searched include Ebscohost (MEDLINE, PsycINFO PsycARTICLES, and Academic Search Complete CINAHL with Full Text). Grey literature repositories were also explored in order to find any non-indexed literature of significance to the scoping review. These included CHW Central; USAID; WHO; One Million Community Health Workers Campaign; Global Health Workforce Alliance (GHWA); and Advancing Partners & Communities (APC). Literature searches were done between January and April 2018.

Title screening of candidate papers was done by one reviewer (LMM). The accepted articles were imported into EndNote library reference management software. Duplicates were removed. The library was then shared between two reviewers for abstract screening. The abstract screening tool was initially piloted on a sample of 10 academic citations to attain reviewer interrater agreement. A kappa interrater agreement was computed to determine the agreement level. Upon establishment of agreement levels, abstracts were screened for eligibility by two reviewers, using the criteria that were determined beforehand, as advised by the pilot test. Consensus was used to iron out disagreements.

### Appraising the quality of evidence

According to Cochrane Collaboration,^46^ a quality filter needs to be applied at some stage in the process of evidence synthesis, and flawed studies must be rejected. Quality of evidence was not conducted since the purpose of the review was to scope the body of literature on CHW programmes and their integration into National Health Systems. This manner of evidence synthesis is provided for under the Arksey and O’Malley^41^ systematic review framework which this study employed. The Arksey and O’Malley framework suggests that study appraisal is optional in scoping reviews.

### Extracting and reporting the data

Sifting and sorting of accepted study components of interest was done using all-inclusive and uniform data extraction forms. Admitted literature were examined for which CHW programmes’ integration aspects they addressed, what conclusions were drawn with respect to the six integration variables, and how the relevant evidence is clarified. These were noted, reviewed and classified across all studies.

The modified version of PRISMA^47^ was used for reporting results. Halas et al.^48^ and Nelson et al.^49^ commended the use of this modified version as best practice for reporting results from scoping reviews. Therefore, the review used the PRISMA checklist, which has been modified to include the components that correspond to the basis of the scoping review methodology, while excluding elements that, for example, are not those that relate to bias. At the heart of the review is thematic analysis of the findings that relate to categories agreed upon beforehand and those evolving during extraction from the included studies. NVivo^50^ helped organise already identified themes according to the conceptual framework.

### Synthesising the evidence

Applying the conceptual framework as presented in Figure 1, the synthesising of evidence was geared towards a general appreciation of how CHW programmes are designed and operated and picking evidence of integration from these processes. This was then refined into a CHW integration narrative. This synthesis resulted in a fine-tuned general understanding of how CHW programmes are interwoven into National Health Systems by finer examination of each of the six CHW programmes in terms of the six integrative variables. At this synthesis stage, the study focused on the integrative philosophy of each CHW programme, looking for strengths and weaknesses among the selected programmes and considering common attributes among them while looking for evidence of differences between stated and actual programme objectives and expectations if carried in any of the available literature.

### Screening results

**FIGURE 2:**
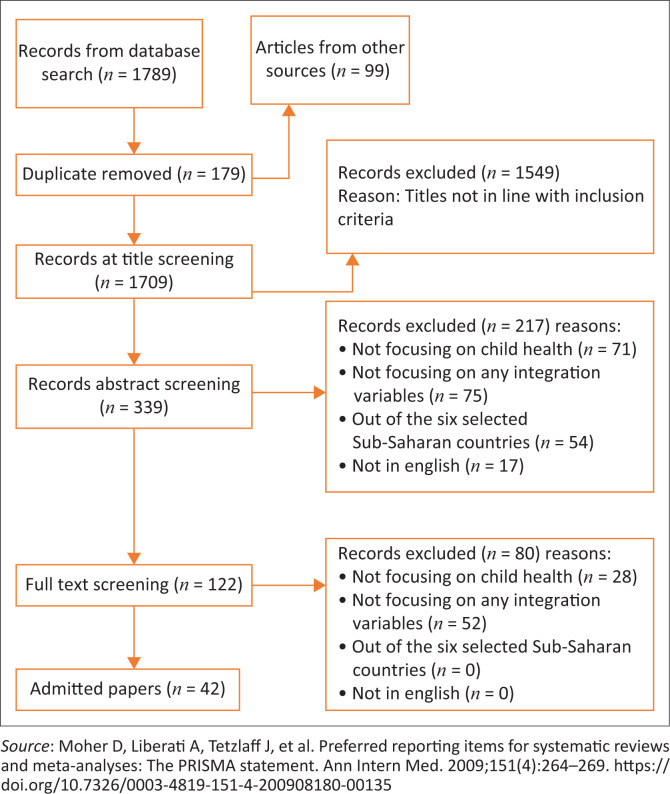
Preferred reporting items for systematic reviews and meta-analyses flow diagram.^47^

A total of 1789 articles were extracted from search databases. Furthermore, 99 additional articles were identified from references to the articles initially found. A total of 786 articles were screened after a total of 158 articles were excluded during full article screening for the reasons indicated in the PRISMA diagram (Figure 2). In the end, 42 articles were admitted for thematic analysis.

## Review findings

This section presents the results of evidence of the integration of CHW programmes into National Health Systems in SSA. The results are divided into three sections: characteristics of reviewed papers, characteristics of the included CHW programmes, and evidence of integration.

### Characteristics of the studies

**FIGURE 3:**
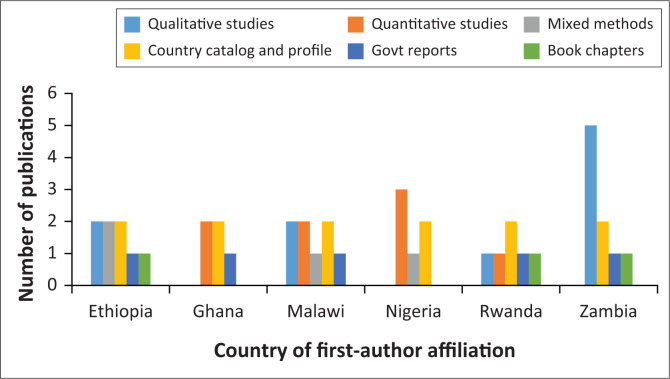
Characteristics of studies included in the study.

Peer-reviewed journals and grey literature repositories that were searched yielded a total of 42 peer-reviewed journal articles and policy documents that were admitted for the final review. Out of these, six were Community Health Systems Catalogues, six were Community Health Programmes Country Profiles, three were Book chapters with case studies, five were National Community Health Strategic Plans, 10 were qualitative studies, eight were quantitative studies and four used mixed methodologies. Figure 3 illustrates type of studies while Appendix 1 shows other comprehensive characteristics of the different studies by the author and year of publication, country, and aim of study.

### Summarised characteristics of the included community health worker programmes

**FIGURE 4:**
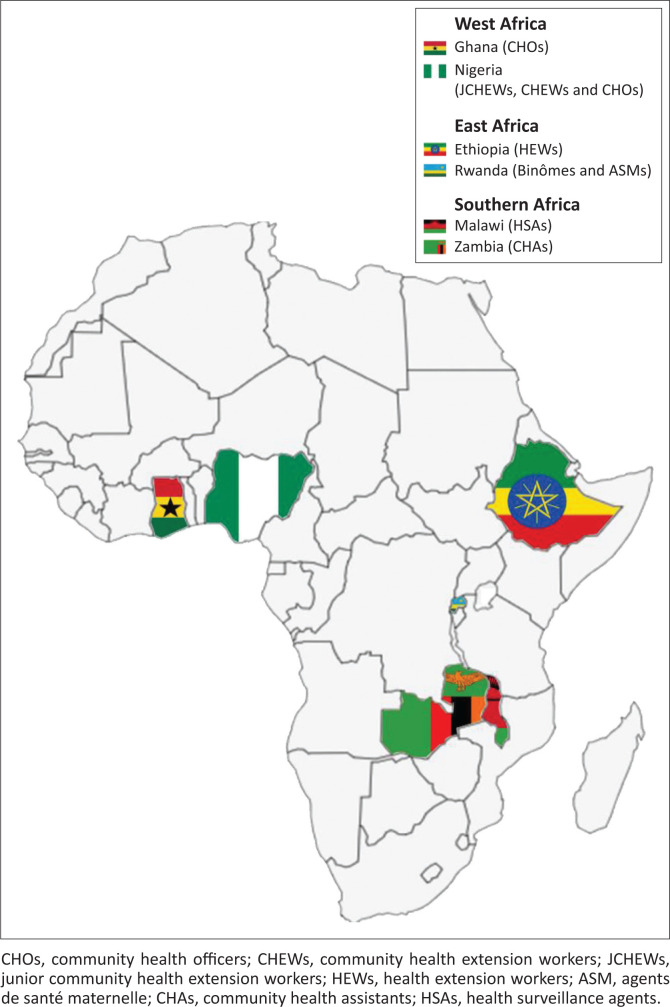
Mapping the selected community health worker programmes.

This section gives an overview of the six national CHW programmes admitted into the study. Ethiopia’s HEWs work under the National Health Extension Programme^57,58,59,66^; Ghana’s CHOs operate under the country’s Community-based Health Planning and Services (CHPS)^65,71,72^; Malawi’s HSAs implement the National Health Surveillance Agent (NHSA) Programme^73,76,77^; JCHEWs, CHEWs and CHOs of Nigeria drive the country’s National (and state) Primary Health Care Development Agency (NPHCDA)^54,78^; Rwanda’s binomes and ASMs work within the National Community Health Programme^51,52,53,66^ and lastly the CHAs of Zambia that function within the National CHW Programme.^64,66,74,75^ Figure 4 maps the selected CHW programmes by region, country, and name of CHWs.

### Key findings on evidence of integration

This section presents key findings of the integration of various CHW programmes in their respective countries, as found in the accepted literature. Based on the evidence, the integration variables or themes of CHW programmes for assessing evidence of the integration of CHW programmes into health systems have been developed inductively. They were then paired with critical functions based on their perceived suitability to strengthen the respective WHO health system building blocks. For each building block of the health system, the review examined whether the integration process was in place, as evidenced by the existence of broader government and/or MOH policies that refer to the merger and inclusion of CHWs in the health system.^38^ The evidence of integration sought for each integration variable of the CHW programme was standardised national guidelines and plans for CHW recruitment, CHW training, CHW documentation and information management, CHW remuneration, CHW supplies and equipment, and CHW supervision, as recommended by the policies. A summary of the characteristics of studies reviewed is presented in Appendix 1. Table 1 summarises the most important country-specific characteristics of CHW programmes, CHW typology (programme name, name of CHWs, year of establishment, number of CHWs and linkage to the health system), and the most important findings on integration in the six components of CHW programmes.

**TABLE 1:** Community health worker programmes’ components in National Health Systems critical functions.

Region	Country	CHW typology and brief history	Linkage to health system	CHW recruitment, education and certification (human resources for health)	CHW roles and responsibility (service delivery)	CHW remuneration (health financing)	CHW supervision (governance and leadership)	CHW information management (health information)	CHW equipment and supplies (medical products and technologies)
East Africa	Ethiopia^57,58,59,60,62,63,64,66^	Ethiopia Health Extension Program; HEWs; established by the government of Ethiopia; launched in 2003; over 38 000 HEWs.	Health extension workers are employed by the Ethiopian government and are connected to government health posts.^57^	Community health workers’ recruitment selection criteria: age, female gender preference, educational level, local residence. Preservice training: 1 year training duration; practical and theory sessions; refresher trainings every 2 years; accredited training curriculum; trained at Technical and Vocational Education and training institutions.	Roles and responsibilities include health promotion, disease prevention, and treatment of uncomplicated and non-severe illnesses such as malaria, pneumonia, diarrhoea and malnutrition. They provide immunisations, injectable contraceptives, basic first aid, diagnosis and treatment of malaria and diarrhoea, and treatment of intestinal parasites.	Paid by the government of Ethiopia Financial incentives: ■ Per diems and salaries Nonfinancial incentives: ■ Formal social recognition, opportunities for career advancement, and benefits such as annual leave. In some areas, community members and local government residences.	Health extension workers are supervised by the medical professionals at the health centres on a weekly basis. They use a checklist to supervise the HEWs. They also receive periodic supervision visits by Woreda District Health Officers for HEWs, often accompanied by an NGO that provides technical supports.	Health extension workers are the first tier of the NHMIS. Use a family folder, a family-centred tool that feeds into the National Health Management Information System.	Health extension workers distribute including natural family planning, condoms, oral pills, implants and oral rehydration salts. They obtain supplies using an official request procedure from the health centre. They register the supplies, drugs and equipment they obtain at the health post. Health extension workers also access emergency backup supplies from the health centre.
	Rwanda^66,51,52,53,82,83^	Community Health Program; ASM, binomes; number of CHWs is 45 011 (14 873 ASMs and 29 746 binomes); the programme began in 1995.	The Community Health Program in Rwanda is led by MOH and supported by various NGOs. All CHWs are volunteers and a part of the national health policy and structure.^52^	Recruitment and selection criteria: Age, literacy, community residence, personal attributes Preservice training Trained by the MOH. Both ASM and biomes receive initial training from the health centre staff. The MOH organises periodic refresher and additional trainings when a new programme or policy is introduced.	The ASM provides mostly MNCH-focused services and links pregnant women to the health centre for deliveries. The binome conducts iCCM, including malaria, diarrhoea, pneumonia, and monitoring for malnutrition, as well as community-based provision of FP. Both cadres also conduct health promotion for hygiene, nutrition, breastfeeding, immunisation, FP and early care-seeking behaviour; directly observed therapy for TB patients; and screening for gender-based violence victims.	The Government of Rwanda (GoR) uses a community performance-based financing system Financial incentives: ■ ASM and binomes both receive per diems and compensation through a community performance-based financing scheme for meeting predetermined service delivery goals. Nonfinancial incentives: ■ ASM and binomes receive a variety of nonfinancial incentives, including membership in the CHW cooperative; t-shirts; umbrellas; formal social recognition; mobile phones; boots; flashlights; identification badges and participation in study tours.	Community-level service delivery in Rwanda is managed and coordinated across the national, district, sector, cell and village levels. For both ASMs and binomes, the health centre in-charge provides administrative oversight while the cell coordinators provide technical supervision. Supervised by community health workers, called ‘cell coordinators’ who are heads of all CHWs.	Agents de santé maternelle and binomes collect data using home visit registers and stock cards. Use cell phones to report data from community level. A community health information system was established to gather and collect health information from the community level to supplement HMIS.	Community health workers are linked to the national supply chain, ensuring a reliable supply of community health commodities. Binomes provide zinc tablets, ORS and malaria treatment for children less than 5 years and cycle beads, condoms and oral pills. Agents de santé maternelle and binomes track commodity use with stock cards and request refills from their cell coordinators. Agents de santé maternelle and binomes collect medical waste in disposal boxes and bring them to the health centre for proper disposal.
Southern Africa	Malawi^55,56,73,76,77,79,80,81^	National HSA Program; HSAs; 10 451 HSAs as of 2013.	Employed by MOH and report to Health Facilities.^77^	Recruitment and selection criteria: Age, education, community membership and personal attributes. Preservice training: ■ Health surveillance assistants are trained for 12 weeks. ■ Receive classroom and practical-based training ■ Health surveillance agent training is done at PHC training centres and selected districts. ■ Training guided by MOH training policies and curricula.	Health surveillance assistants’ responsibilities include health promotion and delivery of services for family planning, HIV, TB, malaria prevention and nutrition. Health surveillance agents collect vital statistics and maintain village household registers.	The MOH and NGOs finance all incentives. Financial incentives: ■ Salaried as a civil servant ■ Also receive per diems and salary top-ups. Nonfinancial incentives ■ Bicycles.	Zonal officers, national programme managers, DHOs, senior HSAs, environmental health officers, district programme coordinators and community health nurses supervise HSAs. Health surveillance assistants’ supervisors monitor HSA activities, assess quality of activities through direct observation, provide support and feedback on performance, and record results of visit and ensure HSAs meet performance objectives.	-Health surveillance assistants collect data using specific forms including the Village Health Register, TBA Card, CBDA Card and VHC forms. -Health surveillance assistants send these forms to the nearest health facility where initial analysis is made. Data are analysed quarterly by the District Health Officer and disseminated to the different MOH and NGO health programmes in the district. A report on community level indicators is also sent back to the HSAs.	Health surveillance assistants are provided with start-up kits including items for water treatment; test materials like sputum collection boxes; buckets; measuring tape; insecticides; job aids and counselling cards. Plans for obtaining emergency backup supplies are not outlined in policy. Community health officers experience regular stockouts of critical medicines due to poor stock management and insufficient funds at the national level.
	Zambia^61,66,67,68,69,70,74,75,84^	National Community Health Worker Program; CHAs; first group of CHAs was trained in 2011 and deployed in 2012.	Community health assistants are employed by the government.^75^	Recruitment and selection criteria: Age, education, community residence and endorsement, women preference Preservice training: ■ Twelve months training comprised of theoretical-based and practical experiences at selected health facilities and communities. ■ Training is guided by nationally accredited curriculum at existing MOH training institutions.	Community health assistants are responsible for health promotion and disease prevention. Roles and responsibilities on child health include referring clients to the health centre for immunisations; organise outreach sessions in the community for immunisation days, identify and refer cases of neonatal sepsis, provide ORS and zinc to children with diarrhoea, recognise signs of and refer cases of pneumonia, diarrhoea with dehydration, measles, cancer, meningitis, mumps, tetanus and leprosy. Administer deworming medication. Promote appropriate complementary feeding for babies. Administer vitamin and/or iron supplementation.	The MOH/government is responsible for incentives of community health workers. Financial incentives: ■ Salary Nonfinancial incentives: ■ Bicycle, mobile phone, shoes, an umbrella, a backpack and a uniform.	Community health assistants are supervised by the in-charge at the nearest health centre. Supervision is conducted at the health post and in the community level on a monthly basis using standardized supervisory checklists. Supervisors are equipped with a supervision manual and monthly supervision tools to facilitate routine supervision.	Community health assistants submit community-level data supervisors at health posts and health centres using activity reports, stock sheets and registers of the number of clients. Health data collection is integrated with data collected by CDAs supporting community development programmes. DMOs compile data from health facilities into reports, which are then passed on to PMOs and then to the The Ministry of MCDMCH. The data are then entered in the NHMIS.	Community health assistants distribute condoms, oral contraceptives, emergency contraceptives, ORS, zinc, malaria treatment and antibiotics for respiratory infections. They are given a start-up package which they replenish stocks according to the stock sheet balances. They bring medical waste to the health facility for disposal.
West Africa	Ghana^65,71,72,85,86^	National Community-based Health Planning and Services (CHPS); CHOs; piloted 1994–1999; rolled out to national implementation in 1999; number of CHNs 15 900.	Community health officers are salaried health workers based at CHPS compounds who deliver the CHPS service package.^72^	Community health workers’ recruitment and selection criteria: Age, education level, personal attributes Two years of training to become CHN certificate. Receive an additional 2-week training on the CHO role. Additional training is provided as needed.	Preventive health: They provide reproductive, maternal and child health services, manage diarrhoea, treat malaria, acute respiratory infections and childhood illness and provide comprehensive family planning and childhood immunisation outreach.	Community health officers are salaried health workers. Ministry of Health is responsible for their incentives. Financial incentives: ■ Salary and place to live within the CHPS compound. Nonfinancial incentives: ■ Bicycles, formal social recognition for their work.	Community health officers are directly supervised by CHMCs and the officer in charge at health centres.	Community health officers should collect data using paper-based forms and submit them to the SDHMT, where they are compiled and passed to the DHMT.	Community health officers carry drugs such as paracetamol; ORS, multivitamin; first-line malaria drugs and contraceptives including injections, oral pills and condoms.
	Nigeria^54,78,87,88,89,90^	Primary health care system: 1. CHOs 2. CHEWs and 3. JCHEWs; 117 568 CHOs, CHEWs and JCHEWs combined.	Community health officers, CHEWs and JCHEWs are government employees, working and connected to government facilities.^54^	Recruitment and selection criteria: ■ Age and education. Preservice training: ■ JCHEWs and CHEWs receive training at state schools of health technology. One year of on-the-job training with mentorship and completing 2–3 years of formal training. ■ Community health officers are trained at a teaching hospital for 2 year of formal training at a school of health technology. ■ Training is guided by accredited curricula. ■ They receive additional training as needed.	Community health extension workers and JCHEWs provide general preventative, curative and prereferral care to the population as the entry point of the health care system at the community level. They also provide IEC across health sectors.	Community health extension workers, JCHEWs and CHOs paid employees of the government of Nigeria. Financial incentives: ■ Salaries Nonfinancial incentives: ■ Not specify in policy	Community health officers are the highest-level cadre at the community level. Community health officers are supervised by the PHC coordinator/director who are a doctor. Community health extension workers are supervised by CHOs. Junior community health extension workers are supervised by CHEWs.	Community health officers, CHEWs and JCHEWs use health maps, house numbering systems, home-based records contraceptive pills (child health cards, personal cards), a facility-based family card, wall charts, health facility district referral forms, health facility registers and other tools to collect and record data. They submit the data forms to the PHC where the information is consolidated into a facility-based summary sheet that is submitted to Local Government Area LGA NHMIS unit. Data collected are an integral part of the existing HMIS.	Community health officers, CHEWs and JCHEWs provide condoms, oral pills, contraceptives and UIDs for family planning. In addition, CHOs distribute zinc, ORS, malaria treatment, immunisations, vitamins and minerals for childhood illness and infectious diseases. They access supplies at the health facility at which they are based. The government provides the supplies,

HEWs, Health Extension Workers; NGO, Non Governmental Organization; ASM, Animatrice de Santé Maternelle; MNCH, Maternal, Newborn and Child Health; FP, Family Planning; HMIS, Health Management Information System; HSA, Health Surveillance Agent; HSAs, Health Surveillance Assistants; DHOs, District Health Officers; HIV, Human Immunodeficiency Virus; TBA Card, Traditional Birth Attendant Card; CBDA card, Community-based Distribution Agents Card; VHC, Village Health Committee; PHC, Primary Health Care; ORS, Oral Rehydration Salts; CDAs, Clinical Document Architectures; MCDMCH, The Ministry of Community Development, Mother and Child Health; PMO, Principal Medical Officer; DMO, Designated Medical Officer; CHAs, Community Health Assistants; MOH, Ministry Of Health; CHOs, Community Health Officers; CHW, Community Health Workers; CHMCs, Community Health Management Committees; SDHMT, Sub-District Health Management Team; DHMT, District Health Management Team; CHN, Community Health Nurse; CHPS, Community-based Health Planning and Services; PHC, Primary Health Care; CHEWs, Community health extension workers; JCHEWs, Junior Community Health Extension Workers; IEC, Information, Education and communication; LGA NHMIS, Local Government Area National Health Management Information System National Health Management Information System; HMIS, Health Management Information System.

Countries integrate different components of CHW programmes into health systems in various ways. The first point of integration that runs across all countries reviewed is the linkage of CHW programmes to respective health systems. This is evidenced by the fact that all CHWs in the respective programmes implement community health initiatives of the respective governments and are all remunerated by governments in different ways.

With regard to CHW recruitment, education and certification, countries’ integration practices differ. The standardisation of recruitment criteria and the selection process vary from country to country. For example, age and education or literacy requirements are set out in policy documents of all countries. Membership in the community is ascribed for all countries except Ghana and Nigeria, female gender preference is expressed for Ethiopia and Zambia, while personal attributes are prescribed for Rwanda, Malawi and Ghana. Policy guidelines for preservice training of CHWs apply to all countries, although modalities such as duration, curricula development, accreditation, and certification differ.

With regard to service delivery, all reviewed countries have policy guidelines on the scope of the CHW’s work, roles and responsibilities. Although CHWs in different countries provide different services depending on different epidemiological trends and public health needs, health promotion and disease prevention services were common in all countries. The remuneration of CHWs is another component of the CHW programme integrated into the health financing building block in the reviewed programmes. All countries have different financial incentives for CHWs, such as salaries and per diems paid by the respective governments. All countries have policy guidelines on nonfinancial incentives, except for Nigeria. Nonfinancial incentives included work aids such as bicycles and mobile phones, work uniforms such as boots and backpacks, preferential access to healthcare, government residences, social recognition by community members and other healthcare providers, to name a few.

Community health worker supervision is a component of the CHW programme, whose integration into the health system is sporadic across the countries reviewed. For example, all countries provide policy guidelines on who CHW supervisors are, depending on the structures of the respective health systems. However, in some countries, modalities, strategies, procedures and supervision processes such as the use of standardised supervision checklists, the frequency of supervision and CHW-supervisor ratios are not integrated into policy guiding documents. Policy documents from Ethiopia, Malawi and Zambia describe the use of a checklist for the exercise.

Community health worker information management is another component that has evidently been integrated into the critical function of health information of the respective countries under investigation. Firstly and most importantly, CHWs’ generated information has been recognised as the first level of the National Health Management Information System (NHMIS) in all countries reviewed. Although each country uses different data collection tools, all countries have standardised data collection tools. In addition, all countries have policy guidelines for consolidating community and facility data before they are submitted to higher tiers of the health system. However, some countries have not provided guidance on the use of processed information for decision-making locally and on improving health outcomes at the community level.

Finally, the CHW equipment and supplies component of the CHW programmes was integrated into the medical products and technologies building block of the system. This was demonstrated by the fact that CHW supplies are linked to the national supply chain of the health system. Other logistics, such as access, restocking and quality audits, varied depending on the services provided. Another indicator of integration that was evident in all countries except Zambia was policy guidance on the safe disposal of medical waste generated by the CHW service. The rest of the results are summarised in Table 1.

## Discussion

We examined what is published on the integration of CHW programmes into National Health Systems with reference to child health in SSA. The scoping review was structured around the six building blocks of WHO health systems and their corresponding components of CHW programmes. The entire discourse on whether CHW programmes are integrated into the National Health Systems was based on evidence of integration at the policy level, as identified in the reviewed literature. Therefore, the discussion revolves around the latest WHO guidelines for health policy and system support to optimise CHW programmes.

### Human resources for health – Community health workers’ recruitment, education and certification

The CHW programme component that addresses the Human Resources for Health (HRH) function in this study is CHW recruitment, education and certification. Evidence of integration from the examined literature included government and/or MOH policies that articulate recruitment, training modalities and certification guidelines. Information on policy-based selection criteria and processes was found for all six countries reviewed, although with variations. Policy guidelines mentioned age, gender, minimum level of education, membership and acceptance by the target group, as well as personal attributes on the selection criteria of the CHWs. Contrary to WHO’s recommendation on selection criteria,^9^ age was prescribed in Ethiopia,^66^ Ghana,^72^ Nigeria,^78^ Rwanda^52,53,66^ and Zambia^61,66,74,75^ with the exception of Malawi. Other studies have shown correlation between the age of CHWs and their ability for record keeping, use of job aids and client satisfaction.^91^ This seems to be the basis of age restrictions for the selection of CHWs in all countries except Malawi. Malawi, however, seems to be in line with WHO’s best practices in terms of age.^9^ Although the female gender of CHWs was linked to effectiveness in client counselling and enablement compared to the male gender,^91^ this was contradicted in other studies that found no relationship between sex and performance.^92^ In this context, Table 2 shows that female gender preference as a selection criterion was a requirement for CHWs in Ethiopia^57,58,59,64,66^ and Zambia^61,74,75^ while gender mix was indicated for Malawi^77^ and Rwanda.^51,52^

Another selection criterion reported in all countries was the level of education. Ethiopia reported 10th grade as a prerequisite^57,58,59,64,66^ and Ghana specified Community Health Nurses training,^65,71,72,85^ Malawi stipulated the Malawi School Certificate of Education,^56,73,77,81^ Nigeria mentions high school education as a prerequisite for entry as a JCHEWs before progression to become CHEWs and CHOs,^78^ Rwanda requires ability to read and write,^51,52,53,66^ and Zambia specified a minimum of a grade 12 education.^61,66,74,75^ Although it has been noted that literate CHWs tend to be younger,^93^ the requirement for literacy seems to be unequivocal among the surveyed CHW programmes. Even though some studies have shown that CHWs with higher educational qualifications have opportunities for alternative employment and therefore migrate from one job to another,^94^ entry level educational qualifications in our results seem to suggest a tendency to recruit those with secondary school education and higher to enhance their effectiveness in executing their CHW duties and deliver better care to the community. Still on selection criteria, local residents as a requirement was reported for Ethiopia,^57,58^ Malawi,^73,76,77^ Rwanda^51,52,53,66^ and Zambia.^61,66,68,70,74^ Lastly, under CHW recruitment, evidence of integration was apparent in policy guidelines on personal attributes as a selection criterion. Varied personal attributes were prescribed for Ghana,^72^ Malawi^77^ and Rwanda.^52^ In line with WHO’s strong contraindication on use of marital status as a CHW selection criterion,^9^ none of the programmes specified it as such.

The WHO suggested the use of certain criteria to determine duration of preservice training. These include: scope of work, anticipated role and responsibilities, competences required for service delivery, prior knowledge and skills institutional capacity, and expected conditions of practice among many others.^9^ The study found that most programmes made use of the above as policy guidelines for standardised CHW training duration varied across the selected country programmes. For instance, initial training duration for Ethiopia’s HEWs^57,59,62,66^ and Zambia’s CHAs^61,66,67,68,69,74,75,84^ is 1 year, Ghana’s CHOs is 2 years,^65,72,86^ Malawi’s HSAs is 12 weeks,^56,76,77^ Nigeria’s JCHEW, CHEWs and CHOs^54,78,87,90^ is 2–3 years, and Rwanda’s ASMs and binomes is around 10 days. Although training duration varied across the six country CHWs under study, study sponsorship was the common denominator among these programmes. This aspect is perhaps the single most direct evidence of CHW integration of the CHW training into National Health Systems. This finding resonates well with other research findings that governments must assure that a fundamental set of skills and information is provided to CHWs, through training, given the extensive role that they CHWs play in primary care.^5,24^

### Service delivery (community health workers’ role and responsibilities)

The CHW programme component that has been identified to improve the service delivery in this study is CHW roles and responsibilities. Evidence of integration abstracted from surveyed literature included government and/or MOH policies articulating guidelines on CHW roles and responsibilities^31^ including their functions, duties and activities of CHWs, as well as their relationship to the health system.^38^ The scope of CHW services in all admitted programmes was clearly defined at policy level. Health promotion, disease prevention and community mobilisation were articulated in policies of all the six countries. Clinical services like treatment of uncomplicated and non-severe illnesses were mentioned for Ethiopia,^57^ Ghana^72^ and Nigeria.^54^ This is in sync with other recommendations that the CHW role should be defined and considered in relation to other health workers.^5,9,24^ Regarding CHW programmes’ relationship with the health system, the surveyed literature showed that CHW programmes from the admitted literature had a pronounced linkage with respective National Health Systems at policy level. These findings resonate with earlier recommendations on the need for a clear linkage between CHW programmes and health systems.^5,24^ For instance, in East Africa, HEWs are employed by the Ethiopian government^57^ and Rwanda’s CHWs implement a programme that is led by the MOH and is part of the national health policy and structure.^52^ In Southern Africa, Malawi’s HSAs are employed by the MOH and report to health facilities^77^ and Zambia’s CHAs are employed by the government.^75^ In West Africa, Ghana’s CHOs are salaried health workers who deliver the CHPS service package,^72^ and CHOs, CHEWs and JCHEWs are government employees, working and connected to government facilities.^54^

### Health financing (community health workers’ remuneration)

In the study, evidence of integration for health financing was government/MOH guidelines, records on CHW remunerations and incentive payments.^31^ That is, the health system provides a balanced incentive package reflecting job expectations, including financial compensation in the form of a salary and non-financial incentives.^38^ This integration variable corresponds with research findings on how remuneration and incentives for CHWs are essential and hence the need for inclusion in the health financing plans of the health systems.^5,24^ The WHO’s guideline on health policy and system support for CHWs strongly endorses remunerating CHWs for their work with a financial package that equals with the job demands, complexity, number of hours, training and roles undertaken.^9^ The study’s findings are in line with these endorsements. For instance, HEWs are employed and paid per diems and monthly salary by the government of Ethiopia^57,58,59,60,62,63,64,66^ and their nonfinancial incentives include formal social recognition, opportunities for career development, annual leave, training, and place of residence in some areas.^57^ Similar findings were observed for Ghana,^58,71,72,85,86^ Malawi,^56,73,76,77,79^ Nigeria^54,78,87,90^ and Rwanda.^51,52,53,66,82,83^

### Leadership and governance (community health workers’ supervision)

Evidence of integration sought for under this integration variable include government/MOH standardised CHW supervision plans and guidelines, supervisor job descriptions and qualifications, supervision checklists or other tools, supervision reports and supervision training documents.^31^ Numerous appraisals of current CHW programmes have documented lack of CHW supervision as a hindrance to delivery of quality community health services by CHWs.^5^ As a result, direct supervision of CHWs by facility-based staff has been commendable to strengthen integration of CHWs into health system.^24^ In particular, WHO’s guidelines on health policy specified a list of supportive supervision strategies that could be employed for CHW programmes. They include appropriate trained supervisors, coaching and mentoring and prioritising enhancing the quality of supervision.^9^ Findings from the review indicate variations for each country. For instance, in Ethiopia, HEWs are periodically supervised by a district medical professional supervisory team comprising a health officer, a public health nurse, an environmental/hygiene expert, and a health education expert.^57,58,59,60,62,63,66^ On the other hand, Ghana’s CHOs are supervised by community health management committee (CHMC) and officer in charge of the health centre or a public health nurse at the district level.^65,71,72,86^ In Nigeria, JCHEWs and supervised by CHEWs, whilst CHEWs are supervised by CHOs, who are in turn supervised by primary healthcare coordinators who are doctors.^54,78,87,89^ Agents de santé maternelle and binomes are supervised administratively by the health centre in charge^52^ and technically by CHWs named cell coordinators and assistant cell coordinators.^51,52,53,66,82,83^

### Information (community health workers’ documentation and information management)

In this study the integration variable that resonates with the information health system function is the CHW documentation and information management. The evidence of integration sought for under this integration variable was that government through MOH and/or health system provides policy guidelines on CHW documentation and information management,^31^ particularly articulating how CHW collected data flows to the health system and back to the community, and how they are used for quality improvement.^38^ The study checked if there are standardised CHW notebooks, recording formats, and record keeping guidelines on how CHWs’ document visits. For instance, Ethiopia’s HEWs are reported to use a family folder containing a family-centred tool that feeds into the NHMIS.^57,58,59^ Ghana’s CHOs collect data that are submitted to the SDHMTs and District Health Management Teams (DHMTs) before they are passed on to the Ghana Health Service.^65,72,71^ In Zambia, HSAs collect community data using specified forms, which they send to the nearest health facilities. The data are analysed quarterly at district level and disseminated to MOH programmes in the district. Health surveillance agents also receive feedback reports on community level indicators.^73,76,77,81^ Nigeria’s JCHEWs, CHEWs and CHOs use prescribed data forms to collect data that are submitted to the primary health centre where the information is consolidated into facility-based summary sheet which ends up as integral part of the existing HMIS.^54,78^ Agents de santé maternelle and binomes in Rwanda collect data using home visit registers and stock cards, which end up at the MOH community health information system which was designed to collect data from community level to the NHMIS.^51,52,53,83^ In Zambia, CHAs use prescribed tools to collect community level data that are submitted to the District Health Information Systems and National Health Systems.^68,70,74,75^ The findings from the programmes harmonise with WHO recommendation regarding CHW data collection and use, that practicing CHWs should document the services that they provide, and that they should also collect and use the health data during their daily undertakings.^9^

### Medical products and technologies (community health workers’ equipment and supply)

Evidence of integration for this variable included national government provision of requisite equipment and supplies to CHWs that are needed to deliver services as expected. Examples include: MOH guidelines for CHW stocks and supplies, supply ordering procedures and forms, inventory forms and procedures.^31^ Research has previously indicated how CHW acceptability in the community is closely linked with commodity availability, and hence the need by the health system to ensure a steady supply of all commodities and material support essential for their duties.^5,24^ Our findings resonate with WHO’s recommendations on the integration of CHW equipment and supply into the overall health supply chain as a way of ensuring adequate availability of commodities.^9^ In Ethiopia, HEWs use official request procedure to get supplies, drugs and equipment from the health centre, which get supplied from the national supply management system.^57,58^ In Ghana, CHOs access commodities from supply stores at CHPS compounds and they are expected to maintain tally cards to track use of medicines and supplies.^65,72^ In Malawi, HSAs are provided with start-up kits that get restocked at local health facilities during management meetings.^73,77^ In Nigeria, the government is responsible for providing supplies to JCHEWS, CHEWs and CHOs through the health facilities at which they are based.^54,58^ In Rwanda, binomes and ASMs access supplies through health centres which are linked to the national supply chain. In addition, they track commodity use with stock cards and request for refills through their cell coordinators.^51,52,53^ Lastly, in Zambia, CHAs are provided with start-up packages of material products and supplies by supervisors at health posts and health centres from the national supply chain.^68,70,74,75^

## Study limitations

This study focused on CHW programmes run by respective governments. This has a limiting bearing on the kind of integration insights that might have been gained were it to also include CHWs not run by respective National Health Systems. The shortcomings of the health system building blocks framework have a limiting effect on the methods used as the study documents were selected based on the building blocks and the abstracted evidence of integration mapped on the same. The study design also limited the kind of recommendations that the study would have delivered. This is because the admitted papers focused on contextual issues surrounding CHW integration at the expense of theoretical explanations for the same. In addition, the study did not assess the feasibility of the policies to indicate if they are likely or unlikely to be put into practice. Lastly, we did not appraise the quality of papers admitted into the study and this could undermine the quality of the findings.

## Conclusion

Globally, CHWs need to complement existing health services to meet unmet community health needs. This has made strengthening health systems to improve child health outcomes a contemporary discourse and priority for health systems today. Zambruni et al.^30^ recounted how universal health coverage (UHC) in all countries is unattainable by 2030 without strengthened community health systems. Although there is no common approach to the integration of CHW programmes, most reviewed literature unanimously agreed on the need to integrate CHW programmes.

As evidenced by the results of this review, some SSA countries have heeded the CHW integration call, but there is still a need to further investigate the extent or degree of integration of CHW programmes into the broader health systems. In large part, this scoping review underscores the willingness of health systems in the region to integrate CHW at the policy level. Evidence of integration through policy-based guidelines for all selected integration variables runs across countries and shows the complexity of CHW integration. Most importantly, the next step, given the implementation guidelines, would be to measure the extent of integration at the implementation level. Rigorous assessments of validated integration measurement metrics are however required in future CHW integration research.

Given the importance of this link to the success of CHW programmes, these study findings could inform policy makers and CHW programme managers on how to strengthen health systems to improve child health outcomes. This could help to further reduce the burden of morbidity and mortality in SSA.

## Appendix 1

**TABLE 1-A1:** Characteristics of studies reviewed.

First author and year	Country	Aims of study
Devlin e t al. 2017^57^	Ethiopia	To provide the most up-to-date information available on community health systems based on existing policies and related documentation in Ethiopia.
Advancing Partners & Communities 2013^58^	Ethiopia	To collect the most up-to-date information available on the community health system, CHWs and community health services in Ethiopia.
Perry et al. 2017^66^	Ethiopia	To provide an overview of large-scale CHW programmes in Ethiopia.
MOH Ethiopia 2015^59^	Ethiopia	To improve equity, coverage and utilisation of essential health services, improve quality of health care and enhance the implementation capacity of the health sector at all levels of the system.
Kok et al. 2015^60^	Ethiopia	To understand how relationships between HEWs, the community and health sector were shaped, in order to inform policy on optimising HEW performance in providing maternal health services.
Lunsford et al. 2015^63^	Ethiopia and Tanzania	To increase HIV testing among pregnant women and antenatal care service utilisation and improve sanitation.
Medhanyie et al. 2012^62^	Ethiopia	To investigate the knowledge and performance of HEWs on antenatal and delivery care. The study also explored the barriers and facilitators for HEWs in the provision of maternal health care.
Damtew et al. 2011^64^	Ethiopia	To examine conditions that may affect the quality of HEWs training in Ethiopia.
Egan et al. 2017^72^	Ghana	To provide the most up-to-date information available on community health systems based on existing policies and related documentation in Ghana.
Advancing Partners & Communities 2013^65^	Ghana	To collect the most up-to-date information available on the community health system, CHWs and community health services in Ghana.
Moh Ghana 2016^71^	Ghana	To attain the goal of reaching every community with a basic package of essential health services towards attaining universal health coverage and bridging the access inequity gap by 2030.
Sacks et al. 2015^86^	Ghana	To examine domains of community health nurse satisfaction and motivation.
Baatiema et al. 2016^85^	Ghana	This analysis presents systematic and comprehensive evidence of CHWs’ contributions and health policy gaps in Ghana.
Devlin et al. 2016^77^	Malawi	To provide the most up-to-date information available on community health systems based on existing policies and related documentation in Malawi.
Advancing Partners & Communities 2014^73^	Malawi	To collect the most up-to-date information available on the community health system, CHWs and community health services in Malawi.
MOH Malawi 2017^76^	Malawi	To ensure quality, integrated community health services which are affordable, culturally acceptable, scientifically appropriate and accessible to every household through community participation. This strategy focuses on multiple areas such as integration of health services; community engagement; sufficient and equitable distribution of well-trained community health workforce; and sufficient supplies, transport and infrastructure.
Gilroy et al. 2012^80^	Malawi	To assess the quality of care provided by HSAs – a cadre of community-based health workers – as part of a national scale-up of CCM of childhood illness in Malawi.
Callaghan-Koru et al. 2013^79^	Malawi	To: (1) describe the types and levels of selected health system supports provided to HSAs performing CCM in Malawi and (2) identify factors that constrain and facilitate the delivery of health system supports.
Lewycka et al. 2010^55^	Malawi	To evaluate two community-based interventions aimed at reducing newborn and infant mortality rates through either mobilisation of women’s groups or home visits by village women volunteers focusing on maternal and infant care and feeding practices.
Kok et al. 2016^56^	Malawi	To obtain in-depth insight into the facilitators of and barriers to interpersonal relationships between HSAs and actors in the community and health sector in hard-to-reach settings in two districts in Malawi, in order to inform policy and practice on optimising HSA performance.
Chikaphupha et al. 2016^81^	Malawi	To explore factors that influence motivation of HSAs in Malawi, with the aim of identifying interventions that can be applied to enhance motivation and performance of HSAs.
Egan 2017^72^	Nigeria	To provide the most up-to-date information available on community health systems based on existing policies and related documentation in Nigeria.
Advancing Partners & Communities 2014^78^	Nigeria	To collect the most up-to-date information available on the community health system, CHWs and community health services in Nigeria.
Findley et al. 2013^88^	Nigeria	To describe early results of an integrated MNCH programme in Northern Nigeria where child mortality rates are two to three times higher than in the southern states.
Ordinioha et al. 2010^89^	Nigeria	This study was performed to report the experience of the use of CHEWs in the provision of primary care in a private rural health care facility in a community in Nigeria.
Uzondu et al. 2015^90^	Nigeria	To explore the feasibility of deploying resident female CHEWs to rural areas to provide essential MNCH services.
Charyeva et al. 2015^87^	Nigeria	To (1) assess the knowledge and skills of trained CHEWs in the provision of implants; (2) determine satisfaction of clients with services provided by CHEWs; (3) assess the extent to which the mechanisms support CHEWs’ provision of implants functioned as intended and (4) determine facilitators and challenges encountered by CHEWs in the provision of implants.
Egan et al. 2017^72^	Rwanda	To provide the most up-to-date information available on community health systems based on existing policies and related documentation in Rwanda.
Advancing Partners & Communities 2013^51^	Rwanda	To collect the most up-to-date information available on the community health system, CHWs and community health services in Rwanda.
Perry et al. 2017^66^	Rwanda	To provide an overview of large-scale CHW programmes in Rwanda.
MOH 2018^53^	Rwanda	To identify gaps and opportunities and lay out policy guidelines with an aim to contribute to the achievement of health targets as identified in the MDGs, Vision 2020 and EDPRS. To provide clear guidance for the provision of holistic and sustainable health care services to communities with their full participation.
Condo et al. 2014^82^	Rwanda	To assess the capacity of CHWs and the factors affecting the efficiency and effectiveness of the CHW programme, as perceived by the CHWs and their beneficiaries.
Drobac et al. 2013^83^	Rwanda	To document the collaboration between the MOH and PIH, in HSS activities that focus on improving health care access, quality, delivery and health outcomes.
Devlin et al. 2016^77^	Zambia	To provide the most up-to-date information available on community health systems based on existing policies and related documentation in Zambia.
Advancing Partners & Communities 2013^74^	Zambia	To provide the most up-to-date information available on the community health system, CHWs and community health services in Zambia.
Perry et al. 2017^66^	Zambia	To provide an overview of large-scale CHW programmes in Zambia.
MoH, Zambia, 2010^61^	Zambia	A National Community Health Worker Strategy has been developed by the MOH with the aim of repositioning and expanding the currently available CHW cadre.
Zulu et al. 2015^68^	Zambia	To demonstrate whether implementation of policy guidelines for integrating community-based health workers in the health system may not automatically guarantee successful integration at the local or district level.
Zulu et al. 2014^84^	Zambia	To explore CHA experiences and how these affect their motivation to do a good job.
Zulu et al. 2013^69^	Zambia	To address the human resources for health shortage and the challenges facing the community-based health workforce in Zambia.
Phiri et al. 2017^70^	Zambia	To explore the facilitators and challenges in implementing the CHA programme, for strategic learning and programmatic improvement.
Shelley et al. 2016^67^	Zambia	The CHA scope of work includes preventive and curative services related to disease prevention and control, behavioural health, environmental health, reproductive health, child health, and medical and surgical conditions.

CHA, community health assistant; CHW, community health worker; MOH, Ministry of Health; HSS, health system strengthening; MDG, Millennium Development Goals; CHEWs, community health extension workers; MNCH, maternal, newborn, and child health; HSAs, health surveillance assistants; HSA, health surveillance agent; CCM, community case management; HEWs, health extension PIH, Partners in Health; EDPRS, Economic Development and Poverty Reduction Strategy.

Note: Please see the full reference list of the article, Mupara LM, Mogaka JJO, Brieger WR, Tsoka-Gwegweni JM. Community Health Worker programmes’ integration into national health systems: Scoping review. *Afr J Prm Health Care Fam Med*. 2023;15(1), a3204. https://doi.org/10.4102/phcfm.v15i1.3204, for more information.
